# CFTR activity in nasal potential difference of adults with idiopathic bronchiectasis

**DOI:** 10.1186/s12931-026-03599-1

**Published:** 2026-03-03

**Authors:** Burkhard Tümmler, Angela Schulz, Rebecca Minso, Nadine Alfeis, Stephanie Tamm, Jessica Rademacher, Felix C. Ringshausen

**Affiliations:** 1 Department of Pediatric Pneumology, Allergology and Neonatology, Hannover Medical School, Carl-Neuberg-Str. 1, Hannover, D-30625 Germany; 2 Department of Respiratory Medicine and Infectious Diseases, Hannover Medical School, Hannover, Germany; 3 Biomedical Research in Endstage and Obstructive Lung Disease Hannover (BREATH), German Center for Lung Research (DZL), Hannover, Germany

**Keywords:** Idiopathic bronchiectasis, CFTR, CFTR-related disorder, Nasal potential difference, Epithelial sodium channel ENaC

## Abstract

**Background:**

Multiple documented underlying etiologies may lead to bronchiectasis, but the European Bronchiectasis Registry found that in 38% of patients the cause is unknown, referred to as idiopathic. We wanted to resolve the role of CFTR dysfunction in people with idiopathic bronchiectasis by nasal potential difference (NPD) measurements.

**Methods:**

NPD was examined in people with cystic fibrosis (CF), healthy controls, 40 people with idiopathic bronchiectasis recruited from the local outpatient clinic and in 60 people with idiopathic bronchiectasis with the suspected etiology of CF who had been referred from 2010 – 2022 to our electrophysiological laboratory to make a diagnosis by NPD.

**Results:**

The unselected MHH cohort and the preselected diagnostic cohort of people with idiopathic bronchiectasis matched in their basal NPD potential with healthy controls. Conversely, after inhibition of the sodium conductance with amiloride, the distribution of the CFTR-mediated depolarization potential upon exposure to chloride-free solution and isoproterenol was in between those of healthy controls and CF patients with exocrine pancreatic insufficiency and overlapped with that of patients with exocrine pancreatic sufficiency. This intermediate phenotype was characteristic for the whole study population of 100 people with idiopathic bronchiectasis irrespectively of whether clinical features of CFTR dysfunction had been recognized before in an individual. Taking the Sermet Score that was developed to discriminate patients with CF from non-CF patients by NPD, the bronchiectasis population was significantly distinct from both healthy people and people with CF.

**Conclusions:**

A CFTR activity of the nasal surface epithelium in the lower quartile is typical for people with idiopathic bronchiectasis, but further CFTR-independent inherited susceptibilities and external insults are necessary to materialize the emergence of bronchiectasis.

**Trial registration:**

Clinical trial number: not applicable.

**Supplementary Information:**

The online version contains supplementary material available at 10.1186/s12931-026-03599-1.

## Background

Bronchiectasis (BE) is a chronic inflammatory lung disease defined radiologically when permanent bronchial dilatation is seen on chest computed tomography (CT) [1, 2]. Multiple documented underlying etiologies may lead to BE [3], but the European Bronchiectasis Registry (EMBARC) found that in 38% of patients the cause is unknown, referred to as idiopathic [4].

Bronchiectasis is also a leading pulmonary symptom in the systemic disorder cystic fibrosis (CF) [5]. This autosomal recessive trait is caused by mutations in the *Cystic Fibrosis Transmembrane Conductance Regulator* (*CFTR*) gene. In the lungs, CFTR conducts chloride and bicarbonate transport in surface-airway epithelia and submucosal glands, thus determining airway surface liquid volume and mucus hydration [6, 7]. Absent or defective CFTR leads to mucus plugging and bronchial obstruction initiating a vicious cycle of infection, inflammation and airway remodeling merging in fibrosis and bronchiectasis.

Full-blown CFTR dysfunction causes the multi-organ disease CF, but residual levels of CFTR function in the gray zone between CF and non-CF are associated with a group of several clinical conditions called CFTR-related disorders (CFTR-RDs), with Congenital Bilateral Absence of the Vas Deferens (CBAVD), recurrent pancreatitis and disseminated bronchiectasis being the most common CFTR-RDs. Recently the European Cystic Fibrosis Society (ECFS) published a series of four papers dealing with the definition and diagnosis of CFTR-RD, assessment of CFTR function with biomarkers and the follow-up and treatment of the specific disorders [8–11]. Accordingly a CFTR-RD is defined by three components, first, the presence of specific clinical features, second, the exclusion of a diagnosis of CF, and third, the evidence of a partially functioning CFTR protein, whose lack of activity does not reach CF thresholds, with in vivo or ex vivo CFTR functional tests [8].

CFTR activity in the airways can be assessed in vivo by the nasal transepithelial potential difference (NPD) across nasal surface epithelial cells [12]. Perfusion with amiloride and subsequently chloride-free/isoproterenol solutions leads to hyper- and depolarization of the basal potential as measures of the sodium and chloride permeability of the epithelial membrane mediated by the epithelial sodium channel ENaC [13] and the chloride channel CFTR [6, 7], respectively. North American and European NPD Standard Operating Procedures (SOPs) were published for using the NPD as a biomarker of CFTR function [12]. To differentiate people with CF from subjects with non-CF by cut-off values of the NPD, two composite diagnostic scores, the Wilschanski score [14] and the Sermet score [15], have been established. The two scores take into account both the hyperpolarization response to amiloride and the depolarization responses to chloride-free/isoproterenol solutions.

The proportion of subjects with CFTR-RD among people with BE is still unknown. Of a single center cohort of 601 adults with BE, 46 subjects were diagnosed with CF (7.6%) [16]. *CFTR* mutation analysis in 91 individuals with idiopathic BE or idiopathic sinopulmonary disease revealed 17 subjects with two sequence variants and 26 carriers of one sequence variant in the *CFTR* gene [17, 18] suggesting that carriers of *CFTR* sequence variants should constitute a large group in the BE population. NPD measurements revealed an anomalous depolarization potential in the CF range in 20 of 68 subjects with idiopathic sinopulmonary disease [18]. Bienvenu et al. have performed the yet largest single center NPD study on individuals with idiopathic BE [19]. The cohort of 122 patients exhibited intermediate values of the Wilschanski score that were significantly different from healthy controls on one side and people with CF on the other whereby median values shifted closer to the CF range in the order of 0, 1 and 2 identified sequence variants in the *CFTR* gene [19].

As a follow-up of the seminal study by Bienvenu and colleagues published in 2010 [19], we revisited the issue of the range of CFTR dysfunction of the respiratory epithelium in people with idiopathic BE. Our site harbors the CF and the BE outpatient clinics under one roof and is the major domestic center to make a diagnosis of cases with inconclusive *CFTR* genetics and sweat test by NPD, intestinal current measurement (ICM) or sweat secretion test (SST) [20, 21]. We hypothesized that individuals with idiopathic BE suspected to suffer from CF or CFTR-RD define a subgroup that is more affected by CFTR dysfunction than the whole group of people with idiopathic BE. To address this issue, we compared the NPD between an unselected cohort of people with idiopathic BE seen at our clinic and a preselected group of individuals with disseminated BE suspected to suffer from CF who had been referred to our CF electrophysiology laboratory to make a diagnosis. Unexpectedly, the two cohorts matched in their NPD features distinct from healthy controls suggesting that subnormal CFTR activity of the respiratory tract is typical for adults with idiopathic BE.

## Material and methods

### Study population

Adults with idiopathic BE who were attending the BE outpatient clinic of Hannover Medical School (MHH) were contacted during the 10-month study period by a study nurse whether they would be interested to be assessed at their next visit for CFTR dysfunction by measurement of their NPD. Data of the current bronchiectasis severity index [22], lung function, airway microbiology, anthropometry, co-morbidities and smoking history were compiled from the digital patient file (see Additional File 1). Subjects were not invited if they could not make the round trip within a day or if they had received a lung transplant, were seriously ill or were carrying multidrug-resistant pathogens in their airways.

The investigated diagnostic cohort of patients with disseminated BE presented symptoms of airway disease and co-morbidities suspicious for CF that alerted the treating physician to ask for an assessment of CFTR dysfunction by NPD at our center. After the first contact had been established in person or by phone or email, the referring physician was asked to substantiate the suspected diagnosis of CFTR dysfunction by providing case history and medical findings for review and to transmit the results of Gibson-Cooke pilocarpine iontophoresis sweat tests and the report of *CFTR* mutation analysis of a certified human genetic laboratory. Data about sweat chloride concentration, *CFTR* genotype, anthropometry, exocrine pancreatic status and features of respiratory, gastrointestinal and hepatobiliary disease were then extracted from the written information (see Additional File 2).

Since cigarette smoke induces CFTR dysfunction, the smokers were assessed by NPD on at least two separate occasions. These persons were requested to refrain from smoking for at least the two weeks prior to the day of NPD measurement. Patients who systemically administered beta blockers or topically applied rhinologicals such as steroids, xylo- or oxymetazoline were requested to stop the medication for the 24 h prior to the scheduled NPD assessment.

Healthy non-smokers with no history of CF among their relatives were recruited from students and staff of MHH.

Of the people with CF who regularly attended the outpatient CF clinic of MHH, patients with exocrine pancreatic insufficiency (PI-CF) or exocrine pancreatic sufficiency (PS-CF) were asked to volunteer for an assessment of their basic defect by NPD.

All study participants or their parental guides provided informed consent. The study was approved by the Ethics Committee of MHH (approval numbers 5391, 6071 and 6790).

### NPD measurements

NPD was measured according to the SOP_EU001, version 1.7 of the ECFS Diagnostic Network Working Group & Clinical Trials Network with the subcutaneous skin bridge as reference and the Marquat double lumen catheter as the nasal exploring bridge. The SOP was slightly modified in-house to improve the reproducibility and stability of the measurements [20]. We expanded the wording for the set-up of stock solutions (see Additional File 5). The make-up of the low-capacity phosphate buffers was modified so that magnesium phosphate does not precipitate, as this affects pH and voltage stability. Second, the handling of the ATP solutions was specified to avoid depurination and dephosphorylation of the nucleotide.

All NPD data reported in the Additional Files 1–3 were post hoc evaluated from the electronically stored digitized tracings by applying the quality and evaluation criteria outlined in the SOPs 528.00 and 529.00 of the CFFT TDN.

### Statistics

Since the Shapiro – Wilk test revealed that all NPD responses were not normally distributed, a Kruskal–Wallis test was applied to the NPD data of the five groups of the study cohort followed by pairwise comparisons with the Two Sample Mann–Whitney U test. Raw P values were adjusted by Bonferroni correction. Calculations were performed with online tools provided by Statistics Kingdom (https://www.statskingdom.com/).

## Results

### Characteristics of the study population

The study cohort consisted of 100 people with idiopathic BE, nine healthy controls, seven people with PS-CF and 15 people with PI-CF. The 40 people with idiopathic BE recruited from the local BE clinic (BE MHH) were significantly older (median age of 62 years, inner quartile range (IQR) 42–70, range 18–79 years) than the 60 people with idiopathic BE with the suspected etiology of CF who had been referred from 2010–2022 to our center to make a diagnosis by NPD (BE diagnostic, median age of 40 years, IQR 21–56, range 6–77 years) (*P* < 0.00001, Z = 4.79, effect size 0.47). The population had more females than males in both the single center (65%) and the diagnostic (60%) group. The clinical metadata of the people with idiopathic BE are compiled in the Additional Files 1 and 2.

### *CFTR* genetics and sweat test

*CFTR* mutation analysis has been performed in 55 of the 60 individuals of the BE diagnostic cohort seen at our CF electrophysiology laboratory (Additional file 2). Thereby the two yet unknown missense variants p.Gly437Iso and p.Val769Iso were identified. Forty-two of the 55 people with idiopathic BE (76%) were harboring no sequence variant classified by the databases CFTR France [23] and/or CFTR2 [24] as a CF-causing mutation, a likely pathogenic variant or a variant of unknown clinical significance (VUS), respectively. Five individuals were carriers of a CF (2) or a VUS (3) sequence variant on one *CFTR* allele. A *CFTR* genotype of two sequence variants was identified in eight individuals, namely two subjects with two VUS variants, three subjects with one VUS and one CF mutation, one subject with one likely pathogenic variant and one CF mutation and two subjects with two mutations classified by the databases in 2025 as CF-causing. The two individuals with the genotypes p.Phe508del/p.Thr1299Iso and p.Gly542Ter/c.3717 + 12191 C > T were diagnosed by their clinical features as PS CF and are now regularly seen at a CF center, whereas the 45-year old subject with the genotype p.Phe508del/p.Asp1152His was diagnosed by us as CFTR-RD in accordance with her normal spirometry and the highly variable clinical presentation of carriers of the p.Asp1152His mutation [25, 26]. She remains to be seen by the referring physician and occasionally consults the local CF clinic.

Sweat chloride concentrations of pilocarpine iontophoresis sweat test were available for 42 people of the diagnostic cohort at the day of NPD assessment (Table 1). Twelve, 24 and 6 individuals had sweat chloride concentrations in the normal, intermediate and CF range, respectively. Conversely, of the 16 people with idiopathic BE attending the MHH clinic who participated in a sweat test, sweat chloride was in the normal range for 13 subjects and in the intermediate range for 3 subjects. Thus, sweat chloride concentration was significantly higher in the diagnostic cohort than among the patients attending our local BE clinic (*P* = 0.0007; Fisher’s exact test).

**Table 1:** Sweat chloride concentrations of sweat tests of 42 people with BE of the diagnostic cohort*

*CFTR* genotype	Normal	Intermediate	CF range
	< 30 mMol/L	30–60 mMol/L	> 60 mMol/L
WT/WT	9	18	5
VUS/WT		1	1
CF/WT	1		
VUS/VUS	1	1	
VUS/CF	1	1	
CF/CF		3	

^*^The outcome of the *CFTR* mutation analysis was classified according to categories of the CFTR France database (https://cftr.chu-montpellier.fr, accessed October 21 st, 2025). *WT* non-disease-causing sequence variant, *VUS* likely pathogenic sequence variant or variant of unclear clinical significance, *CF* disease-causing sequence variant

### NPD measurements

Of the 100 people with idiopathic BE who were recruited for the assessment of their NPD, no reliable measurements were feasible for four participants from our local BE clinic and only one nostril could be explored for 12 people of the diagnostic cohort. The NPD values of each individual with idiopathic BE are listed in the Additional Files 1 and 2. Additional File 3 provides the NPD data for healthy controls, PI CF and PS CF. Table 2 summarizes the outcome for the two BE cohorts, healthy controls and the people with CF. The median values of the tracings taken from the BE cohorts were close to those from healthy controls in the basal potential and the response to amiloride. The median depolarization response upon exposure to chloride-free solution and then to isoproterenol and the median Sermet Score were in between the values for healthy controls on one side and for PI CF and PS CF on the other side.

**Table 2:** Assessment of nasal transepithelial potential difference*

Cohort	no of tracings	Basic potential [mV]	Hyperpolarization	Depolarization	Sermet Score
			Δ amiloride [mV]	Δ(Cl^−^_free_ + iso) [mV]	
Bronchiectasis clinic	69	−12 (−19—−8; −59—−1)	7 (5—10; −9 – 24)	−11 (−20—−5; −49 – 6)	0.87 (0.25–1.74; −0.77 – 4.39)
Bronchiectasis diagnostic cases	99	−16 (−23—−12; −61—−6)	8 (4–12; 1–46)	−12 (−18—−5; −36 – 0)	0.75 (0.16–1.55; −1.2 – 3.26)
Healthy controls	16	−17 (−17 – −9; −49—−3)	7 (7–10; 2–32)	−22 (−33—−10; −59—−8)	1.82 (0.89—2.73; 0.38–5.99)
PS CF	11	−44 (−57—−26; −59—−19)	28 (15–34; 8–37)	−7 (−11—−4; −15—−1)	−0.23 (−1.18 – 0.01; −1.26 – 0.7)
PI CF	24	−36 (−49—−31; −58—−16)	21 (19–30; 9–44)	−1 (−5 – 0; −14 – 1)	−0.98 (−1.45—−0.5; −2.09 – 0.1)

^*^Median (inner quartiles; range)

To resolve the overlap and separation of the NPD between BE MHH, BE diagnostic, healthy controls, PS CF and PI CF, we compared the individual data of the five groups by Mann Whitney two sample rank tests. Table 3 provides raw and corrected *P* values, the Z score and the effect size. The two BE cohorts were matching in the distributions of the hyper- and depolarization potentials and the Sermet Score, but deviated significantly in their distribution of the basal potential. Amongst the diagnostic cohort suspected to be affected by CFTR dysfunction more individuals exhibited a more negative basal potential indicating a below-average apical anion permeability of their upper respiratory epithelium. BE MHH and BE diagnostic overlapped with the healthy controls in basal potential and response to amiloride, but responded with lower depolarization to the exposure with chloride-free solution and isoproterenol indicating that the average ENaC-mediated sodium conductance is normal and that the average CFTR-mediated chloride conductance is subnormal in the two recruited BE groups [19, 20]. Consequently, the distribution of the Sermet Score shifted significantly to the CF range. The two BE cohorts were distinct from people with PI CF in their NPD, but the comparison between BE and PS CF yielded a more differentiated picture. The BE and PS CF groups were significantly different in their distributions of Sermet Score, basal potential and response to amiloride, but overlapped in their cumulative response to chloride-free solution and isoproterenol. This depolarization potential reflects the apical chloride conductance of CFTR-positive nasal epithelial cells [27]. People with PS CF are characterized by residual CFTR activity below the threshold between CF and non-CF. Thus, the BE diagnostic group of patients who had been selected for assessment by NPD because of features suspicious for CF and the unselected group of all people with idiopathic BE attending a single BE center share the same range of subnormal CFTR activity distinct from the range of healthy controls. Within both BE groups more than a quarter of the tracings had a Sermet Score in the CF range (Table 2).

**Table 3:** Mann Whitney two sample rank tests

Comparison of groups		raw *P* value	corrected P	Z score	Effect size
A	basal potential				
BE MHH vs BE diagnostic		0.00152	0.015	3.1714	0.24
BE MHH vs healthy control		0.718	1	0.3606	0.039
BE MHH vs PS CF		2.76E-06	3E-05	4.6878	0.52
BE MHH vs PI CF		8.98E-10	9E-09	6.1265	0.64
BE diagnostic vs healthy		0.0286	0.29	2.1887	0.2
BE diagnostic vs PS CF		1.2E-05	1E-04	4.3782	0.42
BE diagnostic vs PI CF		2.2E-08	2E-07	5.5954	0.5
healthy control vs PS CF		7.75E-05	8E-04	3.9519	0.76
healthy control vs PI CF		7.61E-06	8E-05	4.4758	0.71
PS CF vs PI CF		0.286	1	1.0671	0.18
B	response to amiloride				
BE MHH vs BE diagnostic		0.616	1	0.5011	0.039
BE MHH vs healthy control		0.192	1	1.3056	0.14
BE MHH vs PS CF		3.72E-06	4E-05	4.6265	0.52
BE MHH vs PI CF		2.27E-10	2.3E-09	6.3417	0.66
BE diagnostic vs healthy		0.706	1	0.3772	0.035
BE diagnostic vs PS CF		1.1E-05	1E-04	4.396	0.42
BE diagnostic vs PI CF		3.78E-10	4E-09	6.2629	0.56
healthy control vs PS CF		0.000233	0.0023	3.6804	0.71
healthy control vs PI CF		7.2E-06	7E-05	4.4879	0.71
PS CF vs PI CF		0.845	1	0.1958	0.033
C	cumulative response to (chloride-free solution + isoproterenol)				
BE MHH vs BE diagnostic		0.655	1	0.447	0.034
BE MHH vs healthy control		0.0098	0.1	2.5827	0.28
BE MHH vs PS CF		0.0853	0.85	1.729	0.19
BE MHH vs PI CF		1.31E-07	1.3E-06	5.277	0.55
BE diagnostic vs healthy		0.00476	0.05	1.8231	0.26
BE diagnostic vs PS CF		0.105	1	1.6212	0.15
BE diagnostic vs PI CF		1.85E-08	2E-07	5.6256	0.51
healthy control vs PS CF		0.00256	0.026	3.0161	0.58
healthy control vs PI CF		3.97E-06	4E-05	4.6131	0.74
PS CF vs PI CF		0.00269	0.027	3.0017	0.51
D	Sermet Score				
BE MHH vs BE diagnostic		0.339	1	0.956	0.074
BE MHH vs healthy control		0.0143	0.14	2.451	0.27
BE MHH vs PS CF		9.47E-06	1E-04	4.429	0.5
BE MHH vs PI CF		5.80E-13	6E-12	7.2051	0.73
BE diagnostic vs healthy		0.00433	0.043	2.8529	0.27
BE diagnostic vs PS CF		1.71E-05	1.7E-04	4.2996	0.41
BE diagnostic vs PI CF		3.53E-13	4E-12	7.2727	0.65
healthy control vs PS CF		2.44E-05	2.4E-04	4.2204	0.81
healthy control vs PI CF		4.94E-08	5E-07	5.4536	0.82
PS CF vs PI CF		0.18	1	1.3423	0.21

### Peculiar cases

**Fig. 1:**
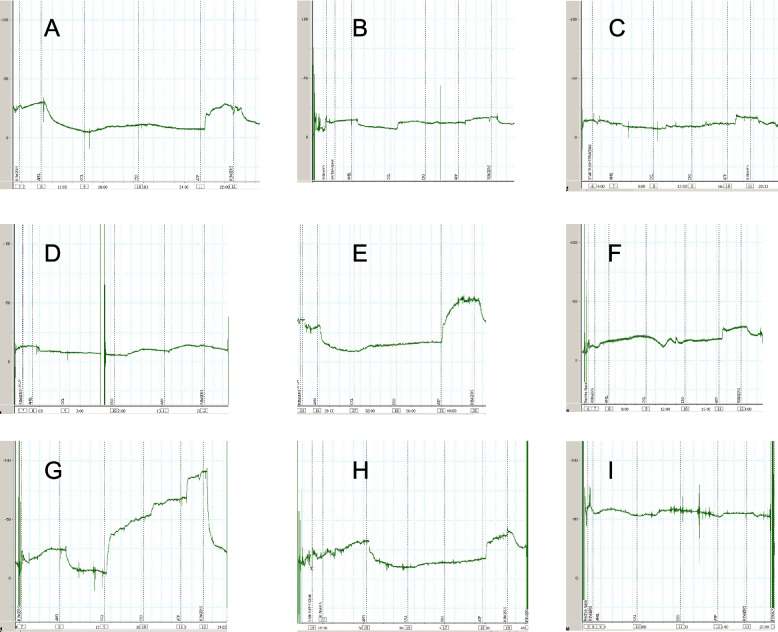
Anomalous NPD in people with idiopathic bronchiectasis seen at the clinic of Hannover Medical School. The original tracings are all displayed to the same scale. Minute depolarization potentials upon exposure to chloride-free solution and isoproterenol are consistent with the diagnosis of CF (**A**) (Additional File 2, subject 41). The tracings **B** and **C** (Additional File 1, subjects 5, 15) are within the gray zone between non-CF and CFTR-RD. No responses upon exposure to amiloride and only subtle responses upon exposure to chloride-free solution and isoproterenol are seen in tracings **F** and **I** (Additional File 1, subjects 6, 37) consistent with the diagnosis of pseudohypoaldosteronism. The tracings **D**, **E**, **G**, **H** show recurrent measurements of the right (**D**, **G**) and left nostril (**E**, **H**) of the same subject 28 recorded at the first visit (**D**, **E**) and 99 days later (**G**, **H**) (Additional File 1, lines 32, 33). Please note concordant responses of the left and highly discordant responses of the right nostril. Nasal examination revealed no macroscopic anomalies

Among our 100 individuals with idiopathic BE the adult with the p.Gly542Ter/c.3717 + 12191 C > T genotype was the only person with the full-blown phenotype of CF. Even the other person classified by us as PS CF exhibited a normal ICM of CFTR-mediated apical chloride secretion in the rectal epithelium indicating that the *CFTR* mutations conferred disease to the respiratory tract (Fig. 1, panel A), but not to the intestine.

Subjects were diagnosed as CFTR-RD if they were carrying one or two non-benign [23] *CFTR* sequence variants and/or showed sweat chloride concentrations in the intermediate or CF range and exhibited a Sermet Score in the CF range. Thereby 19 individuals of the diagnostic BE cohort and two individuals of the BE MHH cohort were allocated to the CFTR-RD group. However, as shown in Fig. 1B and C several subjects generated tracings in the gray zone between CFTR-RD and non-CF consistent with the notion that CFTR activity in the upper airways of people with BE shows a continuum from CF to non-CF [18, 19].

Subject 28 produced NPD tracings in one nostril that were most discordant amongst all tracings we have recorded during the last 30 years at our center. Whereas the tracings were similar in the left nostril on both occasions (Fig. 1E, H), the responses to amiloride and to chloride-free solution and isoproterenol in the right nostril were highly different on two occasions (Figs. 1D, G). Considering that the median intra-subject differences of the hyperpolarization and depolarization responses have been just 2 mV and 4 mV among hundreds of NPD measurement during the last 15 years [20], we suggest that this subject is rather unique in her ability to strongly modify ENaC and CFTR activities over time.

Besides CFTR dysfunction, the tracings of the MHH cohort also uncovered subjects with dysfunction of the sodium channel ENaC. The nostrils of subjects 6 and 37 did not show any response upon exposure to amiloride and only subtle responses upon exposure to chloride-free solution and isoproterenol (Fig. 1F, I). We have previously seen similar NPD tracings in an index case who carries the p.Phe61Leu loss-of-function mutation in the *SCNN1A* gene encoding the alpha subunit of the amiloride sensitive epithelial sodium channel ENaC [28]. Correspondingly, we propose that subjects 6 and 37 are affected by pseudohypoaldosteronism type I that resembles pulmonary features of CF in the absence of common *CFTR* mutations [29–31].

## Discussion

Our NPD measurements revealed that the unselected MHH cohort and the preselected diagnostic cohort of people with idiopathic BE matched in their basal potential with healthy controls. Conversely, after the ENaC-mediated sodium conductance had been inhibited with amiloride, the distribution of the CFTR-mediated depolarization potential upon exposure to chloride-free solution and isoproterenol was in between those of healthy controls and PI CF and overlapped with that of PS-CF. This intermediate phenotype was characteristic for the whole study population of 100 people with idiopathic BE irrespectively of whether clinical features of CFTR dysfunction had been recognized before in an individual. Taking the Sermet Score that was developed to discriminate patients with CF from non-CF patients by NPD [15], the BE population was distinct from both healthy people and people with CF (see Table 3D).

Soon after the cystic fibrosis *CFTR* gene had been identified [32], the geneticists demonstrated a higher frequency of CF-causing mutations among people with idiopathic BE than in the general population [16, 17]. Like in pancreatitis or CBAVD [33], PS mutations that confer some residual CFTR activity were more prevalent in the BE population than the more severe PI mutations. Correspondingly, when *CFTR* mutation analysis was combined with the CFTR biomarker NPD [18, 19], the “measurements demonstrated a wide spectrum of CFTR dysfunction with an overlapping ‘grey zone’ between non-CF and CF individuals” [18]. This continuum of the upper airway electrophysiological phenotype in people with idiopathic BE could be differentiated between the groups of patients carrying no, one or two *CFTR* mutations, the latter being diagnosed as CFTR-RD [19]. Now taking the criterion of a subnormal depolarization potential, we argue that subnormal CFTR activity is common for the upper airways among people with idiopathic BE. A depolarization potential of zero is characteristic for people with CF carrying two loss-of-function class I mutations in the *CFTR* gene [34]. Thus, the depolarization potential reflects the CFTR-mediated chloride conductance of the nasal surface epithelium assessed by NPD.

The BE study population was recognized as a separate entity intermediate between healthy controls and people with CF. If a continuum of subnormal CFTR function in the NPD is typical for people with idiopathic BE, their subdivision into people with CF, CFTR-RD or non-CF will not properly reflect the disease entity. To substantiate this argument, we post hoc re-classified the whole BE study population by the criterion whether an individual had generated a normal or not normal NPD tracing (Additional File 4, Tables S1 and S2). The two groups ‘BE normal NPD’ and ‘BE not normal NPD’ matched in their response to amiloride, but were distinct in their distributions of the basal and depolarization potentials and the Sermet score (Table S2). The tracings of the ‘BE normal NPD’ group overlapped with those of the healthy controls. Conversely, the tracings of the ‘BE not normal NPD’ group overlapped in basal potential and hyperpolarization response with those of healthy individuals and in the depolarization response with those of people with PS CF. Correspondingly, the distribution of the Sermet scores was distinct from those of healthy controls, PI CF and PS CF consistent with our proposal that our investigated BE cohort overlaps in their NPD profile with both health and PS CF. Future studies will hopefully resolve the issue whether or not subnormal CFTR function is a general characteristic of idiopathic bronchiectasis or only affects a subgroup. Our study population does not cover the whole spectrum of BE disease, namely the diagnostic group because of the physician’s suspected diagnosis of CFTR-RD and the MHH group because of the referral of sicker and/or more complex cases to our special outpatient clinic.

Having an inherent focus on CF, the ‘ECFS standards of care’ proposes a classification based on the detection of sequence variants in the *CFTR* gene and cut-off values of the CFTR biomarkers sweat test, NPD and ICM [9]. This consensus document is helpful to make a diagnosis and to guide follow-up and treatment, but does not accommodate the specific features of BE in the context of CFTR biology.

On one hand, the prevalent procedure of *CFTR* mutation analysis was developed to make a diagnosis of CF. It scans the exons and flanking intron sequences, but unless the whole gene is sequenced, it will miss most intronic variants such as c.3874-4522A > G highly prevalent in people with BE [35]. On the other hand, ubiquitous functional non-neutral *CFTR* polymorphisms such as M470V [36, 37] and (TG)_m_T_n_ [36, 38, 39], airway-selective enhancer elements, regulatory variants and long-range interactions in the *CFTR* locus [40–47], and transcription [44, 45, 48–50] and splicing factors [51, 52] binding *in trans* to CFTR mRNA transcript present in all people lead to an extensive variability of CFTR expression and activity that is irrelevant for a modulator-naïve individual with PI-CF, but shapes the diversity of the CFTR phenotype of idiopathic BE. CFTR dysfunction can also be acquired. For example, inflammation, ischemia and remodeling have been shown to reduce the abundance of CFTR protein of the nasal epithelium where NPD is measured [53, 54].

To summarize our findings in a single phrase: A CFTR activity of the nasal epithelium in the lower quartile is typical for people with idiopathic bronchiectasis, but further CFTR-independent inherited susceptibilities and external insults are necessary to materialize the emergence of bronchiectasis.

## Supplementary Information

Additional file 1. BE MHH cohort. Clinical metadata and NPD data of people with idiopathic bronchiectasis attending the bronchiectasis clinic of Hannover Medical School.

Additional file 2. BE diagnostic cohort. NPD data, clinical metadata, sweat chloride and *CFTR* genotype of people with idiopathic bronchiectasis referred to the CF Electrophysiology Laboratory of Hannover Medical School for NPD diagnostics.

Additional file 3. NPD data of healthy controls and of people with cystic fibrosis who volunteered to be assessed at the CF Electrophysiology Laboratory of Hannover Medical School to generate in-house reference data.

Additional file 4. Post-hoc classification of the whole study population of people with idiopathic bronchiectasis by the outcome of NPD. NPD data and Mann Whitney two sample rank tests.

Additional file 5. In-house protocol for the set-up of solutions for NPD measurements.

## Data Availability

The dataset supporting the conclusion of this article is included within the article and the Additional files 1–5. The primary NPD data are compiled in the Additional files 1, 2 and 3. Files of the original tracings are available from the corresponding author on reasonable request.

## Abbreviations

BE: Bronchiectasis
CBAVD: Congenital bilateral absence of the vas deferens
CF: Cystic fibrosis
CFFT TDN: Cystic Fibrosis Foundation Therapeutics, Inc., Therapeutics Development Network
CFTR: Cystic fibrosis transmembrane conductance regulator
CFTR-RD: CFTR- related disorder
ECFS: European Cystic Fibrosis Society
EMBARC: European Multicenter Bronchiectasis Audit and Research Collaboration
ENaC: Amiloride sensitive epithelial sodium channel
ICM: Intestinal current measurement
MHH: Medizinische Hochschule Hannover
NPD: Nasal potential difference
PI: Exocrine pancreatic insufficiency
PS: Exocrine pancreatic sufficiency
SCNN1A: Sodium channel epithelial 1 subunit alpha
SOP: Standard Operating Procedure
VUS: Variant of unclear clinical significance
