# Supplementary material for: CFTR activity in nasal potential difference of adults with idiopathic bronchiectasis
Source: Respir Res. 2026 Mar 3;27:160. doi: 10.1186/s12931-026-03599-1 (PMC13064372; doi:10.1186/s12931-026-03599-1)
Supplement: Supplementary file 4 — Additional file 4. Post-hoc classification of the whole study population of people with idiopathic bronchiectasis by the outcome of NPD. NPD data and Mann Whitney two sample rank tests. [file 12931_2026_3599_MOESM4_ESM.docx]

Additional File 4.

Table S1. Assessment of nasal transepithelial potential difference*

Cohort no of Basic potential [mV] Hyperpolarization Depolarization Sermet Score

tracings Δ amiloride [mV] Δ(Cl^-^_free_ + iso) [mV]

Classification prior to NPD

Bronchiectasis clinic MHH 69 -12 (-19 - -8; -59 - -1) 7 (5 - 10; -9 – 24) -11 (-20 - -5; -49 – 6) 0.87 (0.25 – 1.74; -0.77 – 4.39)

Bronchiectasis diagnostic cases 99 -16 (-23 - -12; -61 - -6) 8 (4 – 12; 1 – 46) -12 (-18 - -5; -36 – 0) 0.75 (0.16 – 1.55; -1.2 – 3.26)

Post-hoc classification by the outcome of NPD

Bronchiectasis: not normal NPD 56 -22 (- 31 - -12; - 61 - -1) 8 (4 – 13; -9 – 46) -5 (-7 - -4; -49 – 6) 0.12 (-0.18 – 0.24; -1.2 – 1.39)

Bronchiectasis: normal NPD 112 -14 (-18 - -10; -31 - -6) 7 (5 – 10; 1 – 24) -16(-23 - -10; 0 – -36) 1.29 (0.7 – 1.96; -0.3 – 3.26)

Healthy and CF controls

Healthy controls 16 -17 (-17 – -9; -49 - -3) 7 (7 – 10; 2 – 32) -22 (-33 - -10; -59 - -8) 1.82 (0.89 - 2.73; 0.38 – 5.99)

PS CF 11 -44 (-57 - -26; -59 - -19) 28 (15 – 34; 8 – 37) -7 (-11 - -4; -15 - -1) -0.23 (-1.18 – 0.01; -1.26 – 0.7)

PI CF 24 -36 (-49 - -31; -58 - -16) 21 (19 – 30; 9 – 44) -1 (-5 – 0; -14 – 1) -0.98 (-1.45 - -0.5; -2.09 – 0.1)

*median (inner quartiles; range)

|  | |  |  |  |  |
| --- | --- | --- | --- | --- | --- |
| Table S2. Mann Whitney two sample rank tests: Post-hoc classification of BE patients by the outcome of NPD | | | |  |  |
|  |  |  |  |  |  |
| comparison of groups | | raw P value | corrected P | Z score | effect size |
|  |  |  |  |  |  |
| A | basal potential | |  |  |  |
|  |  |  |  |  |  |
| BE normal vs BE not normal | | 0.0002408 | 0.0024 | -3.6719 | 0.27 |
| BE normal vs healthy control | | 0.4117 | 1 | -0.821 | 0.071 |
| BE normal vs  PS CF | | 3.25E-7 | 3.3E-6 | 5.1083 | 0.45 |
| BE normal vs PI CF |  | 7.16E-12 | 7.2E-11 | 6.8544 | 0.58 |
| BE not normal vs healthy | | 0.02097 | 0.21 | -2.3085 | 0.26 |
| BE not normal vs PS CF | | 0.0005572 | 0.0056 | 3.4516 | 0.4 |
| BE not normal vs PI CF | | 0.00004754 | 0.00048 | 4.0674 | 0.44 |
| healthy control vs PS CF | | 7.75E-05 | 7.8E-04 | 3.9519 | 0.76 |
| healthy control vs PI CF | | 7.61E-06 | 7.6E-05 | 4.4758 | 0.71 |
| PS CF vs PI CF |  | 0.286 | 1 | 1.0671 | 0.18 |
|  |  |  |  |  |  |
| B | response to amiloride | |  |  |  |
|  |  |  |  |  |  |
| BE normal vs BE not normal | | 0.28 | 1 | 1.0803 | 0.082 |
| BE normal vs healthy control | | 0.2641 | 1 | -1.1168 | 0.098 |
| BE normal vs PS CF |  | 0.000001544 | 0.000015 | -4.8054 | 0.43 |
| BE normal vs PI CF |  | 6.888E-12 | 6.9E-11 | -6.86 | 0.59 |
| BE not normal vs healthy | | 0.7578 | 1 | -0.3083 | 0.035 |
| BE not normal vs PS CF | | 0.00007281 | 0.00073 | -3.9669 | 0.12 |
| BE not normal vs PI CF | | 5.332E-8 | 5.3E-7 | -5.4399 | 0.59 |
| healthy control vs PS CF | | 0.000233 | 0.0023 | 3.6804 | 0.71 |
| healthy control vs PI CF | | 7.2E-06 | 7.2E-05 | 4.4879 | 0.71 |
| PS CF vs PI CF |  | 0.845 | 1 | 0.1958 | 0.033 |
|  |  |  |  |  |  |
| C | cumulative response to (chloride-free solution + isoproterenol) | | | | |
|  |  |  |  |  |  |
| BE normal vs BE not normal | | 5.951E-14 | 6E-13 | 7.5107 | 0.58 |
| BE normal vs healthy control | | 0.1432 | 1 | 1.4638 | 0.13 |
| BE normal vs PS CF |  | 0.0009502 | 0.01 | -3.3049 | 0.3 |
| BE normal vs PI CF |  | 7.854E-13 | 7.9E-12 | -7.1637 | 0.61 |
| BE not normal vs healthy | | 2.474E-7 | 2.5E-6 | 5.1597 | 0.6 |
| BE not normal vs PS CF | | 0.1253 | 1 | 1.5331 | 0.18 |
| BE not normal vs PI CF | | 0.00001102 | 0.0001 | -4.3961 | 0.47 |
| healthy control vs PS CF | | 0.00256 | 0.026 | 3.0161 | 0.58 |
| healthy control vs PI CF | | 3.97E-06 | 4E-05 | 4.6131 | 0.74 |
| PS CF vs PI CF |  | 0.00269 | 0.027 | 3.0017 | 0.51 |
|  |  |  |  |  |  |
| D | Sermet Score | |  |  |  |
|  |  |  |  |  |  |
| BE normal vs BE not normal | | 4.441E-16 | 4E-15 | 8.917 | 0.69 |
| BE normal vs healthy control | | 0.2048 | 1 | -1.2681 | 0.11 |
| BE normal vs PS CF |  | 3.802E-7 | 4E-6 | 5.0786 | 0.46 |
| BE normal vs PI CF |  | 2.864E-14 | 3E-13 | 7.6038 | 0.65 |
| BE not normal vs healthy | | 1.139E-8 | 1E-7 | -5.7086 | 0.67 |
| BE not normal vs PS CF | | 0.005982 | 0.06 | 2.7488 | 0.33 |
| BE not normal vs PI CF | | 2.464E-9 | 2E-8 | 5.9638 | 0.66 |
| healthy control vs PS CF | | 2.44E-05 | 2.4E-04 | 4.2204 | 0.81 |
| healthy control vs PI CF | | 4.94E-08 | 5E-07 | 5.4536 | 0.82 |
| PS CF vs PI CF |  | 0.18 | 1 | 1.3423 | 0.21 |
