# Supplementary material for: CFTR activity in nasal potential difference of adults with idiopathic bronchiectasis
Source: Respir Res. 2026 Mar 3;27:160. doi: 10.1186/s12931-026-03599-1 (PMC13064372; doi:10.1186/s12931-026-03599-1)
Supplement: Supplementary file 5 — Additional file 5. In-house protocol for the set-up of solutions for NPD measurements. [file 12931_2026_3599_MOESM5_ESM.docx]

Additional file 5

In-house protocol for the set-up of solutions for NPD measurements

Background: The SOPs issued by CFF TDN and ECFS DNWG were instrumental in the past to increase the accuracy of NPD measurements. Intra- and inter-site and intra- and inter-operator variability have been significantly decreased. However, we noticed during hands-on training sessions of operators that the set-up of solutions is not described in sufficient detail. This statement still applies to the latest video-supported description of the ‘ECFS CTN & US CFF TDN SOP Standardized Measurement of NPD’ published in September 2018 [1].

NPD tracings often showed systematic drifts or very small responses to ATP which we could ascribe to shifts of pH during measurement and degraded nucleotide, respectively. According to this experience a lab manual was written to explain the set-up of solutions in more detail.

The following text follows word-for-word section 3.2. ‘Solutions’ of SOP NPD_EU001, version 1.7. We changed a few words and added practical hints to ensure stability of chemicals and solutions and accuracy of concentrations, pH and buffer capacity. In particular, practical advice is provided to increase the accuracy of the concentrations and to avoid depurination of ATP and the precipitation of magnesium phosphate which will further reduce the inherently low capacity of the phosphate buffer and hence stability of pH. All additions to the SOP and changes in wording are highlighted in blue.

We optimized the handling to gain physicochemical and microbiological stability without changing the composition of the final solutions suggesting that in contrast to a recent proposal [2] we do not need to change the formulation.

**3.2 Solutions**

Abbreviated Solution Solution Contents

Name Letter

Basal Basal solution A Buffered salt solution

Amil Basal + amiloride B 0.1 mM Amiloride HCl in solution A

Low Cl^-^ Zero Cl^-^ + amiloride C 0.1 mM Amiloride HCl in Stock

Zero Cl^-^ solution

Iso Zero Cl^-^ + amiloride + D 0.01 mM Isoproterenol HCl in

isoproterenol solution C

ATP Zero Cl^-^ + amiloride + E 0.1 mM ATP in solution D

Isoproterenol + ATP

**3.2.1. PREPARATION OF THE SOLUTIONS**

1. Solution A and stock zero Cl^-^ solution are prepared using semi-sterile technique and filtered with a 0.22 µm filter. The result is an aseptic product (not sterile).

2. Solutions A, B, C and zero Cl^-^ solution may be refrigerated up to 3 months or frozen at -20°C for up to 6 months.

3. Solutions D and E are freshly prepared within 1 hr prior to use. All solutions should be prepared using semi-sterile technique.

4. Fill five 50cc syringes with the different solutions A, B, C, D and E. Additional syringes may be prepared as needed.

NB1. Amiloride is light sensitive, store in dark.

NB2. Isoproterenol is light sensitive and sensitive to oxidation: after 5 hrs fridge 4% decay.

NB3. ATP is light sensitive, rapidly hydrolyzed in water at room temperature and depurinated during storage at -20°C. Hence, aqueous ATP stock solution must be stored at -80°C for maximal up to six months.

**BASAL SOLUTION (SOLUTION A)**

The base solution is ddH_2_O

Salts MW mM g/L

NaCl 58 148 8.58

CaCl_2_ •2 H_2_O 147 2.25 0.33

KCl 75 4.05 0.30

K_2_HPO_4_ 174 2.4 0.42

KH_2_PO_4_ 136 0.4 0.05

MgCl_2_ • 6 H_2_O 203 1.2 0.24

1. 10 x salt stock solution F: Dissolve 1.65 g CaCl_2_ •2 H_2_O, 1.5 g KCl and 1.2 g MgCl_2_ • 6 H_2_O in 500 mL ddH_2_O at room temperature
2. 100 x KP_i_ buffer stock solution G: Dissolve 0.5 g KH_2_PO_4_ and 4.2 g K_2_HPO_4_ in 100 mL ddH_2_O at room temperature
3. Dissolve 8.58 g NaCl in 500 mL ddH_2_O at room temperature in a laminar floor hood, add 100 mL solution F and 390 mL ddH_2_O.
4. Add 10 ml solution G in 20 aliquots of 0.5 mL under stirring.
5. Check pH (should be 7.4)
6. Filter with 0.22 µm filter
7. Label each container and refrigerate

**STOCK ZERO CL^-^ SOLUTION**

The base solution is ddH_2_O

Salts MW mM g/L

Na Gluconate 218 148 32.26

Ca Gluconate 430 2.25 0.97

K Gluconate 234 4.05 0.95

K_2_HPO_4_ 174 2.4 0.42

KH_2_PO_4_ 136 0.4 0.05

MgSO_4_ • 7 H_2_O 246 1.2 0.24

1. Dissolve 32.26 g Na Gluconate, 0.97 g Ca Gluconate, 0.95 g K Gluconate and 0.24 g MgSO_4_ • 7 H_2_O in 990 mL ddH_2_O at room temperature.
2. Add 10 ml solution G in 20 aliquots of 0.5 mL in a laminar floor hood under stirring.
3. Check pH (should be 7.4)
4. Filter with 0.22µm filter
5. Label each container and refrigerate

Solutions B and C contain amiloride that dissolves slowly in aqueous solution. Set up of stocks of solutions B and C at least 2 days prior to NPD measurement is recommended. The final concentration of amiloride should be 30 mg/L. Weighing should be as exact as possible. Weigh twice about 30 mg amiloride with a precision scale that is accurate within at least ± 0.1 mg. For example, if you weigh 29.7 mg, set up a solution of 990 mL.

**Amiloride (SOLUTION B)**

1. Weigh x mg amiloride with x close to 30 mg
2. Obtain x/30 • 1000 mL solution A
3. Add x mg amiloride at room temperature in a laminar floor hood.
4. Dissolve amiloride under stirring
5. Check pH (should be 7.4)
6. Label each container and refrigerate

**Low Cl^-^  (SOLUTION C)**

1. Weigh y mg amiloride with y close to 30 mg
2. Obtain y/30 • 1000 mL Stock Zero Cl^-^  solution
3. Add y mg amiloride at room temperature in a laminar floor hood.
4. Dissolve amiloride under stirring
5. Check pH (should be 7.4)
6. Label each container and refrigerate

**Isoproterenol (SOLUTION D)**

1. Make a 0.01 mM isoproterenol HCl solution:
2. Take 100mL solution C to make 50 mL solution D and 50 mL solution E at once
3. If use of 1mg/5 mL ampoules of isoproterenol HC

1 M = 248 mg/ml; 0.01 mM = 0.00248 mg/mL = 0.25 mg / 100 mL

Thus, add 0.25 mg or 1.25 mL of isoproterenol to 100 mL solution C

1. Cover syringe with aluminum foil or otherwise to protect isoproterenol from light
2. Label each syringe and use within 1 hr of preparation

**STOCK ATP**

1. Prepare a 50 mM stock solution:
   1. M ATP = 551 mg/mL; 50 mM ATP = 55.1 mg in 2 mL ddH_2_O
2. Make 20 aliquots of 0.1 mL
3. Label each aliquot by date and freeze at -80°C (up to 6 months)

**ATP (SOLUTION E)**

1. Prepare a 0.1 mM ATP solution:

0.1mM ATP = 0.0551 mg/mL = 5.51 mg / 100 mL = 2.76 mg / 50 mL

1. Take 50 mL of solution D
2. Add 1 aliquot with 2.76 mg ATP = 0.1 mL stock to 50 mL of solution D
3. Cover syringe with aluminum foil or otherwise to protect isoproterenol from light
4. Label each syringe and use within 1 hr of preparation

Reference

[1] Solomon GM, Bronsveld I, Hayes K, Wilschanski M, Melotti P, Rowe SM, Sermet-Gaudelus I. Standardized Measurement of Nasal Membrane Transepithelial Potential Difference (NPD). J Vis Exp. 2018;(139). doi: 10.3791/57006.

[2] Sadou Yayé H, Caudron E, Sermet-Gaudelus I, Nicolis I, Prognon P, Pradeau D. Towards harmonization of solutions used for cystic fibrosis diagnosis by nasal potential difference measurements: A formulation approach with CHESS® software. Ann Pharm Fr. 2022;80:26-34. doi: 10.1016/j.pharma.2021.05.002.
